# Hot-Water Immersion (HWI) or Ice-Pack Treatment (IPT) as First Aid for Human Envenomation by Marine Animals? Review of Literature

**DOI:** 10.3390/toxins16060273

**Published:** 2024-06-15

**Authors:** Łukasz Niżnik, Karolina Jabłońska, Michał Orczyk, Martyna Orzechowska, Judyta Jasińska, Barbara Smoliniec, Agnieszka Hućko, Piotr Kosowicz, Anna Klocek, Paweł Słoma, Aleksandra Roztoczyńska, Joanna Toporowska-Kaźmierak, Kamil Jurowski

**Affiliations:** 1 Department of Regulatory and Forensic Toxicology, Institute of Medical Expertise, Łódź, ul. Aleksandrowska 67/93, 91-205 Łódź, Poland; 2 Toxicological Science Club ‘Paracelsus’, Institute of Medical Sciences, Medical College, Rzeszów University, Al. mjr. W. Kopisto 2a, 35-959 Rzeszow, Polandak127010@stud.ur.edu.pl (A.K.);; 3 Laboratory of Innovative Toxicological Research and Analyses, Institute of Medical Sciences, Medical College, Rzeszów University, Al. mjr. W. Kopisto 2a, 35-959 Rzeszow, Poland

**Keywords:** hot-water immersion, ice packs, cold water, first aid, marine envenomation

## Abstract

Envenomation by marine animals poses a significant health concern globally, affecting both local residents and tourists in coastal regions. The primary objective of this review is to critically evaluate the existing scientific literature to determine the most effective first-aid treatment for envenomations caused by marine animals, specifically whether hot-water immersion (HWI) or ice-pack treatment (IPT) provides the best immediate care. This comprehensive review covers a wide range of marine envenomations, from jellyfish stings to stingray injuries. While our focus is primarily on the efficacy of HWI and IPT, we also explore the role of cold-water treatment as a result of its relevance and similarity to ice-pack applications. In addition, we examine other treatments mentioned in the literature, such as medications or vinegar, and highlight their findings where applicable. To provide a clear and structured overview, we summarised the articles in separate tables. These tables categorise the type of research conducted, the marine species studied, the region of origin of the marine species, and the key findings of each study. Our analysis of the available evidence indicates a general consensus in the scientific community on the effectiveness of HWI or IPT for envenomation by marine animals. However, when treating those injuries, it is crucial to consider all factors since there is no universally superior treatment due to the diverse nature of marine habitats.

## 1. Introduction

Envenomation by marine animals is a prevalent risk for individuals involved in water-based recreational activities, like swimming, surfing, and snorkelling. However, it significantly impacts professional and artisanal fishermen, making them the primary victims [1,2,3]. It is crucial to emphasise that marine animal envenomation is a global issue that affects both local populations and unaware travellers. With increasing globalisation and easy access to coastal areas, it has become more important than ever to raise awareness of envenomation caused by marine animals and provide education on preventive measures and first-aid treatment. Neglecting to address this issue can lead to substantial injuries, human morbidity, and, occasionally, mortality [4], along with considerable healthcare expenses. Hence, it is crucial to tackle this problem on a global scale. From a medical perspective, this issue extends from the lack of adequate toxinology education to the knowledge and skills of healthcare professionals who may not be adequately prepared to handle such incidents. Toxinology is the scientific field dedicated to the examination of venoms and toxins generated by a variety of organisms [5]. There are a handful of courses available that touch on certain aspects of clinical toxinology, some with restricted syllabi, others with a minor emphasis on clinical aspects, or with a narrow geographical scope rather than a global perspective [6,7]. Therefore, there is a pressing need to bridge this gap in medical education and training to improve patient outcomes and reduce the global burden of envenomation by marine animals.

There is no doubt that human envenomation caused by marine animals is an actual problem, but not all current recommended first-aid treatments have been proven, though most have a solid basis and appear effective [8]. The symptoms of envenomation can range from mild local reactions to life-threatening systemic manifestations [9,10,11], depending on the type of marine organism involved and the severity of the envenomation [12,13,14,15,16]. These symptoms may subside, but the toxin present in the victim’s body can still cause delayed complications, which are often underestimated but may manifest themselves at a later time, such as in multiple organ dysfunction. This condition is called delayed jellyfish envenomation syndrome (DJES) [17]. Other potential consequences described in the literature include acute renal failure (ARN) caused by anemone sting [18]. Therefore, appropriate first aid is crucial to human health in such incidents. Treatment of this envenomation is often focused on the alleviation of symptoms and prevention of further complications. The approach to a treatment of envenomation depends primarily on the species that cause it [10,12,13,14,19]. These approaches include the use of vinegar or even medications [20]. However, the two most common first-aid methods for marine envenomation are hot-water immersion (HWI) and ice-pack treatment (IPT). HWI involves immersing the affected body part in hot water (usually 40 °C to 50 °C) for approximately 15–20 min, while IPT involves applying a cold compress, with the lowest possible temperature that the victim can withstand to the affected area. Both of these interventions are believed to provide pain relief, reduce inflammation, and promote tissue healing. Other than that, it is worth noting the significance of cold-water therapy because of its pertinence and likeness to the use of ice packs. However, the optimal first-aid treatment remains controversial, and there is a lack of consensus among healthcare professionals regarding the use of immersion in hot water or ice-pack application as first-aid measures for envenomation by marine animals [21,22]. There are definitive studies on the efficacy of hot water in the treatment of fish-related accidents [23]. However, regarding the use of cold water, uncertainties remain, particularly in cases involving cnidarians [24]. Therefore, the purpose of this review is to examine the scientific literature on the use of immersion in hot water and the application of ice packs as first-aid measures for human envenomation caused by marine animals.

Specifically, this review will evaluate the effectiveness and safety of these interventions, as well as the potential risks and benefits associated with each approach.

By critically evaluating the existing literature, this review aims to provide healthcare professionals and people at risk for human envenomations caused by marine animals with a complete understanding of the available first-aid options for this common toxinology problem [25].

In this review, we do not concentrate on specific marine organisms. However, we included several of the most extensively studied ones:

- Jellyfish are divided into three main classes, Scyphozoa, Hydrozoa, and Cubozoa, with Cubozoa being the most dangerous [26]. Jellyfish are equipped with nematocysts in their tentacles, which inject venom, causing pain, swelling, burning, itching, and activates receptors in nociceptive neurones. Cnidocysts are structures found in Cnidaria, traditionally divided into three categories: nematocyst, spirocyst, and ptychocyst [19]. Nematocysts, the most diverse, serve defensive and predatory functions. Spirocysts immobilise prey in anthozoans, while Ceriantharia uses ptychocysts to create protective tubes. Contact with the skin of jellyfish triggers the immediate release of cnidocysts, capable of penetrating human skin and delivering toxic venom.
- The Scorpaenidae family includes several fish species, such as scorpionfish (Scorpaeninae), lionfish (Pteroinae), and stonefish (Synanceinae). Among these, the venom of the stonefish is the most toxic [27]. Stonefish inhabit the shallow waters of the Indo-Pacific region. Each stonefish has 13 dorsal spines, and each spine is connected to a pair of poisonous glands. Stonefish venom contains a powerful membrane-damaging, heat-labile toxin, capable of releasing the substance P and cyclooxygenase, which causes swelling and severe pain. Despite this, in some parts of Asia, such as Hong Kong, stonefish is considered a delicacy [28].
- Weever fish belongs to the Trachinidae family. They possess venomous dorsal spines that cause stinging injuries when stepped on, making them a hazard in sandy and muddy seabed areas along the Mediterranean and European coasts, especially during the summer season [29].
- Stingrays, a subgroup of cartilaginous fish, are part of the Chondrichthyes class. Stingrays have flattened bodies with pectoral fins fused to their heads. The mouth is located on the ventral side. The most dangerous part of a stingray is its tail, which contains a one- to four-spine stinger [30,31].
- Sea anemones, part of the order Actiniaria, are highly diverse marine creatures found at various depths and latitudes divided into two suborders, Anenthemonae and Enthemonae [32]. Sea anemones use venomous stings to catch prey and defend themselves. Although most species pose little threat to humans, some have highly toxic venoms that can be dangerous [11]. Although envenomation caused by sea anemones is not frequent and severe, they constitute a unique example worthy of consideration in this work.

This review underscores the critical need to address the gap in the current literature on first aid for envenomations caused by marine animals. Despite the global prevalence and potential severity of such injuries, there is a surprising dearth of comprehensive reviews that analyse the efficacy and safety of common first-aid treatments such as HWI and the application of IPT. The significance of this topic is enhanced by the increased accessibility to coastal regions and the popularity of water-based recreational activities, which, in turn, increases the risk of encounters with venomous marine organisms. This work not only highlights the urgency of increasing awareness and education on this topic but also serves as a crucial call to action for further research. By synthesising existing knowledge and identifying areas lacking sufficient study, this review aims to foster a deeper understanding among healthcare professionals and the general public, thus contributing to improved prevention, first aid, and management strategies for envenomations by marine animals. This, in turn, will help reduce the incidence and severity of such injuries, ultimately improving public health and safety in coastal areas throughout the world. We recognised that the effectiveness of various treatments might be influenced by different variables. Therefore, we included Table 1 and Table 2 to summarise the HWI and IPT treatment in regard to the examined factors, origins, and the species investigated in the studies, provided this information was available from the authors.

## 2. Results

### 2.1. Hot-Water Immersion as First Aid for Human Envenomation Caused by Marine Animals

#### 2.1.1. Jellyfish

Evidence of the efficiency of HWI in the treatment of envenomation caused by jellyfish is provided in several review studies [22,33], emphasising its superior clinical results and absence of adverse effects. However, considerations of temperature safety and maintenance remain relevant. Numerous studies and case reports have investigated the efficacy of hot-water treatment for jellyfish envenomations. Some studies focus on providing protocols and guidelines for jellyfish sting treatment, advocating for the use of HWI and vinegar [34,35]. There are studies focused on comparing various treatment methods for jellyfish envenomation. The efficacy of heat, papain meat tenderizer paste, and vinegar for *Carybdea alata* stings was examined, finding that immersion in hot water was the most effective initial treatment, resulting in significantly lower pain scores compared to alternative treatments [36]. The efficacy of hot water at 45 °C in comparison to other treatments, such as ice, vinegar, and aluminium sulphate, in terms of pain relief was also proven. Hot water was found to be the most effective, while vinegar was the least effective [37]. Furthermore, a comparison between hot and cold chemical compresses for box jellyfish stings revealed that hot packs were more effective in reducing pain scores and the cessation of pain [38]. Another study compared cortisone cream with hot-water immersion for jellyfish stings, determining that hot water was more effective in treating acute pain and itch [39]. A study compared hot water immersion to cold packs for relieving *Physalia* sp. sting pain. After 10 min, 53% using hot water reported pain reduction compared to 32% with cold packs. After 20 min, 87% using hot water felt relief compared to 33% with cold packs [40]. An important method for evaluating the effectiveness of a given treatment is through case studies, which simulate real-life situations. Little et al. conducted a case study that included two males suffering from jellyfish stings caused by *Carukia barnesi* and *Chironex fleckeri*. Notable pain relief was observed without the need for additional medication. Furthermore, a man stung by *C. fleckeri* reported increased symptoms as the water temperature cooled, which were alleviated by adding hot water [41]. Yoshimoto et al. demonstrated the efficacy of warm baths and compresses in reducing the pain of jellyfish stings, particularly in cases of Irukandji syndrome [42]. In a study conducted by De Donno et al. [43], the authors examined jellyfish sting incidents in a coastal area of Southern Italy, particularly focussing on epidemiology, severity, and treatment protocols. During a five-year period (2007–2011), a total of 1733 jellyfish envenomations occurred. In particular, non-pharmacological treatments, such as rinsing with ammonia, saline solution, alcohol, or chlorine-based disinfectants, were common. Hot water, cold water, and ice packs were also used, although less frequently.

#### 2.1.2. Stonefish

Although controlling conditions such as the water temperature in real-life situations can be challenging, in vitro studies can offer valuable insights into potential treatments for the envenomation of various marine organisms. This is due to their ability to control experimental conditions. Barnett et al. conducted an in vitro study to investigate the efficacy of heat deactivation on the venom of *Synanceia horrida* for potential first-aid applications. The cytotoxicity of the venom was evaluated using human cardiomyocyte cell lines. The results indicated significant suppression of venom cytotoxicity above 39 °C, with an optimal treatment temperature of 42 °C for 20 min suggested by the researchers [44]. These findings, which demonstrate the efficacy of heat against stonefish venom in vitro, have been supported by multiple case studies. A series of eight clinical cases of stonefish poisoning was documented, emphasising the use of warm-water immersion as first aid to alleviate pain and reduce hospitalisation time [45]. Darlene and Phee-Kheng reported a case of stonefish envenomation treated effectively with hot-water immersion, supporting the hypothesis of venom protein denaturation through heat treatment [46]. Tang et al. highlighted the importance of administering antibiotics along with hot-water baths to prevent the proliferation of harmful pathogens, such as *Vibrio vulnificus*. Monitoring the patient condition after treatment was also emphasised [28].

#### 2.1.3. Lesser Weeverfish

According to Emerson, HWI is considered the most effective treatment for lesser weevers (*Echiichthys vipera*), as high temperature deactivates heat-labile proteins, enzymes, and kinins present in the venom of marine organisms. The water temperature should be as high as possible for the patient for up to 30 min [47]. Abdul Jalil and Qayyum warned about potential tissue burns from using HWI as first aid for lesser weever fish envenomation. The authors described a case involving a 16-year-old girl who suffered skin burns on her toe, with a recovery period of up to 9 months, indicating that the water temperature should be warm but not scalding [29].

#### 2.1.4. Stingray

Clark et al. reviewed 119 stingray envenomation cases over 8 years. Group I (100 patients) received hot-water immersion within 24 h for pain relief; Group II (19 patients) reported after 24 h without pain and received no treatment. Of the 74 patients in Group I, who only received hot-water immersion, 65 experienced pain relief without additional analgesics. The study supports hot-water immersion as an effective pain treatment for stingray stings [48]. Myatt et al. assessed stingray envenomation treatment in southern California. The study involved 22 individuals evaluated on different days after the incident. Treatment methods included immersion in hot water alone or preceded by immersion in hot water with povidone-iodine. Patients treated solely with hot-water immersion exhibited fewer persistent symptoms and complications compared to those who received both hot-water immersion and povidone-iodine, although not statistically significant. Pain scores on a 1–10 scale decreased from an average of 7.36 before to 2.18 after immersion in hot water [49].

#### 2.1.5. Lionfish

Resiere et al. conducted a study demonstrating that hot-water immersion (35–40 °C for 60 min) significantly reduces pain from lionfish stings. Envenomation symptoms such as swelling and intense pain were relieved. The mechanism involves the degradation of heat-sensitive proteins and acetylcholine in the venom. However, the authors emphasise that hot-water immersion should be supplemented with additional treatments, including painkillers, antibiotics, and tetanus toxoid, to address potential complications [50]. Toxicology facilities in Poland documented 14 instances of injuries from lionfish (*Pterois volitans* L.) kept in household aquariums within a span of three years. These injuries exclusively affected the victims’ hands, leading to severe pain and systemic symptoms observed in 11 patients. Treatment methods included immersion in hot water, wound cleaning, antibiotic administration, and tetanus prevention. Furthermore, three cases involving exposure to tank-bred lionfish were identified, where typical signs of envenomation were absent, possibly attributed to “empty stings”, where no toxin was released into the victims’ bodies [51].

#### 2.1.6. Scorpionfish

Twenty-three instances of various scorpionfish species (*Scorpaena plumieri* Bloch, 1789 and *Scorpaena brasiliensis* Cuvier, 1829) injuries were documented, all affecting male fishermen. These incidents took place in two separate colonies: the Z-10 Colony located in Ubatuba, São Paulo State (with 17 patients), and the Rio Vermelho Colony in Salvador, Bahia State (with 6 fishermen). The treatment approaches varied, and six patients found relief from pain through immersion in hot water. Other methods used by the patients themselves included systemic painkillers, applying urine to the affected area, using alcohol, and using garlic, but these proved to be largely ineffective [52].

#### 2.1.7. Catfish

During an 8-year period in various regions of Brazil, 127 injuries were documented. The patients initially experienced severe pain and later faced complications, such as infections and retention of bony fragments. The immersion of affected extremities in hot water was used in 20% of the patients and was proven beneficial. Despite this treatment, secondary infections and foreign body reactions remained common [3]. In another study, conducted by Satora et al. [23] in Poland, 17 cases of envenomations caused by *Clarias gariepinus* (Burchell, 1822) and *Heteropneustes fossilis* (Bloch, 1794) have been recorded between 2003 and 2007. These envenomations resulted in symptoms, such as intense pain, numbness, dizziness, local swelling, and redness. HWI was used as a pain control treatment. Another case of *H. fossilis* envenomation in Poland was described, while a 17-year-old fish breeder sought treatment for a minor injury to his right fifth finger from a stinging catfish while cleaning his tank. Despite stable vital signs, he experienced numbness, dizziness, and intense pain. The medical tests did not show major problems aside from an ECG finding. The Poison Information Centre was consulted, and the wound was treated with lidocaine, material removal, and thorough cleansing. In particular, the affected hand was immersed in hot water (45 °C) for approximately 45 min to inactivate venom in the wound [53]. Dorooshi reported two cases of freshwater catfish stings in 2009 and 2011 at the Noor and Ali Asghar Hospital of the Isfahan University of Medical Sciences. Both incidents involved individuals immersing their hands in catfish aquariums, resulting in wounds. Treatment involved immersion of the affected limbs in hot water (temperature below 50 °C), which relieved pain in 30 min. In addition, systemic antibiotics and tetanus toxoid were administered to prevent infection, and wounds were irrigated and left open to heal with secondary intention [54].

#### 2.1.8. Unspecified/Several Organisms

There is evidence supporting hot-water immersion for non-life-threatening marine envenomation cases, advocating for temperatures up to 45 °C and duration based on pain reduction [21]. Several studies investigated the efficacy of HWI in treating pain from marine envenomation. Lau et al. conducted a telephone survey in Hong Kong hospitals, finding that maintaining water temperature during immersion is crucial to prevent recurring pain and proposed solutions, such as optimizing water containers and using isolators like plastic wrap or aluminum foil [55]. Todd and Edsell highlighted the effectiveness of HWI in Mediterranean species sting management, recommending immersion in water not exceeding 45 °C for up to 2 h alongside pain relievers and saltwater wound cleaning [56]. Habib et al. conducted a study at a district hospital in Indonesia, noting that immersion in hot water, as recommended by international guidelines, effectively reduced pain from various marine species poisonings, including lionfish and jellyfish, possibly due to protein and enzyme degradation in venoms [57].

Summarization of examinated studies, regarding HWI is provided in the Table 1.

### 2.2. Ice Packs as First Aid for Human Envenomation Caused by Marine Animals

#### 2.2.1. Jellyfish

Several reviews and research studies provide insights into the management of jellyfish stings with the utilization of cold water or IPT, highlighting the effectiveness of various treatments, depending on the species of jellyfish involved. The use of iced seawater or cold packs (as opposed to freshwater) exhibits a strong analgesic effect by reducing the spread of the venom, thereby functioning as a pain reliever [12]. Furthermore, the application of vinegar (5% acetic acid) is a beneficial procedure for all instances of cnidarian stinging [58]. Lakkis et al. emphasises that the efficacy of warm and cold water in treating jellyfish stings depends on the type of jellyfish and the characteristics of the sting. Warm water is generally effective for alleviating pain and symptoms, particularly for stings causing local pain and redness. It is beneficial for stings not associated with heat-neutralizable toxins. On the other hand, cold water may be more suitable for severe systemic reactions, helping to reduce swelling and inhibit the spread of toxins, especially with jellyfish such as *Chironex fleckeri* [59]. A review by Cegolon et al. aimed to improve the treatment of jellyfish stings, emphasising the importance of tailoring treatment to the species involved. The approach to jellyfish stings is designed to alleviate the local effects of venom, prevent the further release of nematocysts, and control systemic reactions, including shock. In severe cases, the key step is stabilisation and the maintenance of vital functions. To alleviate pain after a *Physalia* sting, hot water can be applied to the affected area of the skin. Ice packs can be effective analgesics in very severe cases of *Carybdea rastoni* stings. First aid for pain relief after *Carybdea alata* stings includes a bath in hot water. The temperature ranges proven to be effective in studies are 40–41 °C or 45 °C. Ice packs and warm water are used to relieve pain after *Carukia barnes* stings. For *Chironex fleckeri*, *Chiropsalmus quadrigatus*, and *Chiropsalmus quadrumanus* stings, hot water at a temperature of 43 °C or higher is applied; heat reduces all venomous activities, making venom less toxic the longer it is exposed to heat. Although hot water appears to be an effective analgesic, its use can be questioned in the case of *C. fleckeri* venom. Applied heat can increase blood flow, leading to an intensification of cardiotoxic effects, opposite to cold, which lowers blood flow, thus limiting venom spread. Stings from *Pelagiidae* (*Pelagia noctiluca*) and *Cyaneidae* (*Cyanea capillata*) are treated with ice packs [12]. Yanagihara and Wilcox compared commonly recommended practices, such as washing with seawater or using ice packs. These approaches can exacerbate venom-induced haemolysis. The author suggests rinsing with Sting No More^®^ Spray or vinegar instead. Heat application at 45 °C for 45 min is recommended as optimal treatment after carefully plucking tentacles, as it significantly reduces venom activity [24]. Currie and Jacups summarised treatment for *Chironex fleckeri* stings, suggesting initial relief with vinegar followed by ice, with additional analgesia required in 48% of cases [60]. In a trial conducted by Isbister et al., ice packs and warm-water immersion for *Chironex fleckeri* stings were compared, finding no significant differences in pain relief or systemic effects between the two treatments [61]. The results obtained by Edelist et al. on jellyfish stings in the Eastern Mediterranean Sea indicated that out of 267 total cases of jellyfish stings, 31 were treated with ice. Among these, 21 showed improvement, while 10 reported worsening or no improvement [62].

#### 2.2.2. Hydroids

In an in vitro study conducted by Rifkin et al., despite the effectiveness of methylated spirit products in dislodging polyps of *L. philippinus*, the authors suggested that rinsing the injured body part, followed by applying cold, should be the optimal first-aid treatment due to its availability [63].

#### 2.2.3. Sea Anemones

Ballesteros et al. examined a severe toxic reaction to an *Anemonia viridis* sting in a 35-year-old oceanographer. Conventional treatments exacerbated symptoms due to the absence of specific first-aid protocols. Common rinse solutions were evaluated, with vinegar and ammonia causing immediate and significant discharge of cnidocysts. Baking soda and freshwater also activated cnidocysts to a lesser extent. Seawater emerged as the only neutral solution and is recommended for post-sting rinsing, indicating the need for further research on sting-specific first-aid protocols. The removal of the tent, followed by washing with seawater and applying ice packs, is suggested by the authors [19].

Summarization of examinated studies, regarding IPT is provided in the Table 2.

## 3. Discussion

The main goal of our study was to review the scientific evidence to answer the question of whether hot-water immersion or ice pack treatment is more suitable in the first-aid treatment of envenomation caused by marine animals. One of the main challenges we had to overcome, and which we want to underscore, is the fact that due to the variability in individual responses to marine stings, a one-dimensional approach to treatment is clearly inadequate. Factors, such as the severity of the sting, the anatomical location of the injury, and the species that caused the trauma, significantly influence the efficacy of the chosen treatment method. Furthermore, in instances of real-life envenomation, affected individuals or witnesses might be under considerable shock or stress, potentially impairing their ability to identify the species responsible for the envenomation and determine the appropriate course of treatment. Therefore, an informed decision about the most appropriate course of action must be based on a comprehensive evaluation of these variables, ensuring that the chosen intervention is optimally aligned with the specific circumstances of the envenomation case.

In real-life situations, it is not always possible to identify the cause of the envenomation or to control the exact temperature of the water; therefore, the results obtained under laboratory conditions are less vague than the results obtained from proactive engagement in the field, which involves direct observation of the victims. It is also worth highlighting that when analysing these two methods of treating that envenomation, we evaluate them based on their pain relief capabilities, which may not always target the underlying mechanisms of action but rather focus on alleviating local symptoms and providing overall anaesthetic effects. It is also important to acknowledge that the description of pain sensation and the effectiveness of pain relief methods are subjective experiences. Consequently, accurately interpreting the scale of pain can be challenging. Therefore, it is important to recognise that when determining the best approach to treating these types of injuries, all factors must be considered. Treating envenomation caused by marine animals should take into account several factors, including individual physiological differences, the specific marine organism involved, and the timing of the incident. However, in real-life scenarios, treatment must also be fast and readily accessible.

In the existing literature, future research endeavours should prioritise the execution of robust clinical studies. Such studies are imperative for elucidating definitive conclusions about the efficacy of HWI or IPT as first-aid measures for marine animal envenomation. Furthermore, there is a pressing need for comparative studies that evaluate the effectiveness of HWI versus IPT in various envenomation scenarios, considering factors, such as the type of marine animal, the severity of the envenomation, and the demographics of the patient. Additionally, longitudinal studies tracking patient outcomes after HWI or IPT interventions are warranted to comprehensively assess both short-term and long-term effects. Moreover, exploring the potential synergistic effects of HWI or IPT alongside traditional pharmacological interventions could offer valuable information on optimising treatment protocols for cases of human envenomation caused by marine animals.

## 4. Materials and Methods

### 4.1. Literature Search Methods

The search for relevant publications on the treatment of envenomation caused by marine species involved the use of the main scientific search engines, including PubMed and Google Scholar. In the process of selecting scientific data, diverse combinations of keywords were used. These included terms such as ‘marine envenomation’ followed by ‘hot’ and ‘hot water’ and ‘ice’ and ‘ice packs’ and ‘cold’ and ‘cold water’ followed by ‘treatment’ and ‘first aid’. The initial selection of the studies involved the evaluation of the titles and abstracts to quickly determine their relevance to the topic. The studies identified as potentially relevant were then subjected to a next step, where a comprehensive evaluation of the complete texts was performed. This thorough examination allowed for a detailed assessment of content, methodology, and conclusions and guaranteed the inclusion of relevant and reliable information.

### 4.2. Data Presentation

For appropriate readability, we classified the information into main paragraphs, which are as follows: (1) hot water and (2) treatment with ice packs/cold water. Additionally, each paragraph was divided into sub-paragraphs, depending on the organism that caused the envenomation. If the provided article described both treatment strategies, we opted to place it in a paragraph where the conclusions highlighted the superior strategy. For a clear and organised overview, we summarised the reviewed articles in separate tables.

## Figures and Tables

**Table 1:** Hot-water immersion therapy summary.

Jellyfish					
Reference	Research Type	Species	Region	Examined Factor	Findings
[22]	Review	Unspecified	Unspecified	Evidence for the use of heat or ice in the treatment of cnidarian envenomations, with the aim of defining which one is more effective	Most studies endorse the use of HWI for pain relief in cnidarian envenomation
[33]	Systematic review	Unspecified	North America and Hawaii	Pain relief after treatment with hot water, vinegar, papain	Hot water exhibited the most significant analgesic effect among the analysed methods
[34]	In vivo and ex vivo studies	*Cyanea capillata*	For in vitro tentacles of *C. capillata* were collected from Puget Sound. For ex vivo, live *C. capillata*, were collected from Dublin Bay	Several methods of first aid protocols in case of *C. capillata* envenomation	The best first aid for *C. capillata* is rinsing with vinegar or Sting No More^®^ commercial product and 40-min-long treatment with HWI (45 °C)
[35]	Review	Unspecified	Singapore	The article contains appropriate steps to take in the event of a jellyfish sting	HWI is recommended for pain relief in jellyfish stung case
[36]	Randomised controlled trial	*Carybdea alata*	Honolulu, Hawaii	Pain perception (VAS scale) after treatment of HWI, papain or vinegar	In 92% of cases, the lowest VAS score for heat was lower than for either papain or vinegar
[37]	Uncontrolled trial	*Carybdea* sp.	Australia	Pain perception after different treatments (ice, vinegar, aluminium sulfate, and hot water at 45 °C)	Hot water proved to the best at pain reduction and its recurrence
[38]	Randomised controlled trial	*Carybdea alata*	Waikiki, Hawaii	Pain relief after treatment with cold and hot compresses	Hot packs treatment provided with approximately 5 times higher chance of pain cessation within 15 min, than control group
[39]	Clinical trial	*Cyanea capillata*	Gothenburg, Sweden	Comparison of pain relief between HWI and topical corticosteroid assessed on a Numeric Rating Scale (0–10)	HWI is more effective for treating acute pain than cortisone up to 6 h after treatment
[40]	Randomised controlled trial	*Physalia physalis*	Australia	Pain perception (VAS scale) after HWI and cold pack treatment	At the 20-min mark, 87% of the hot water group displayed clinically reduced pain compared to 33% treated with ice packs
[41]	Case study	*Carukia barnesi Chironex fleckeri*	Australia	Pain relief	Neither individual required additional pain relief medication after HWI
[42]	Retrospective review	Unspecified	Western O’ahu, Hawaii	Pain relief	The effectiveness of a heat treatment in reducing pain in cnidarian envenomation was higher compared to the use of painkillers or benzodiazepines
[43]	Case study	Unspecified	Coastal arena of Southern Italy	Various treatment	Hot and cold water along with ice packs were utilised, although not so frequently
**Stonefish**					
**Reference**	**Research Type**	**Species**	**Region**	**Examined Factor**	**Findings**
[28]	Case report	Stonefish (*Synanceja horrida*)	Hong Kong, China	Pain relief, and treatment of potential bacterial growth and further necrotizing fasciitis development	Hot water may accelerate bacterial growth and necrotizing fasciitis. Therefore, antibiotics should be administered
[44]	Experimental in vitro studies	Stonefish (*Synanceia horrida*)	Venom collected from James Cook University, Cairns, Queensland, Australia	Cytotoxicity of *S. horrida* venom on human cardiomyocyte cell line at various temperature and incubation times	Incubation period of 20 min at 42 °C is optimal for treating stonefish wounds
[45]	Case series report	Stonefish (*Synanceia* sp.)	Singapore, Myammar, China	Pain relief	In 7 of 8 cases HWI was used to reduce pain. Presumably due to HWI patients staying less time in hospital
[46]	Case report	Stonefish (*Synanceja horrida*)	Kota Kinabalu, Malaysia	Pain relief after HWI conducted after diclofenac and morphine treatment	Pain reduced in scale from 8/10 to 1/10. No further treatment was required after HWI treatment
**Little Weeverfish**					
**Reference**	**Research Type**	**Species**	**Region**	**Examined Factor**	**Findings**
[29]	Case study	Lesser weever fish (*Echiichthys vipera*)	Mediterranean and European coastal areas	Risk of burn after HWI treatment	Thermal burn can occur after HWI treatment
[47]	Review	Lesser weever fish (*Echiichthys vipera*)	Coastline of the UK	Pain management	HWI is considered the most effective marine envenomation treatment
**Stingray**					
**Reference**	**Research Type**	**Species**	**Region**	**Examined Factor**	**Findings**
[48]	Retrospective review	Unspecified	California, USA	Pain relief after HWI or medicaments	Out of 74 patients, who exclusively underwent HWI, 65 achieved pain relief without requiring supplementary therapy
[49]	Observational Study	Unspecified	California, USA	Pain relief, Ongoing symptoms or complications	HWI presumably is more effective than HWI/povidone-iodine
**Lionfish**					
**Reference**	**Research Type**	**Species**	**Region**	**Examined Factor**	**Findings**
[50]	Case series report	*Pterois volitans*	French West Indies	Pain duration and intensity reduction	Immersing the stung limb in hot water can decrease the duration of pain and mitigate potential complications
[51]	Case reports	*Pterois volitans*	Poland	Treatment methods encompassed hot water immersion, wound cleaning, antibiotic administration, and tetanus prevention	HWI is method utilised by toxicology facilities
**Scorpionfish**					
**Reference**	**Research Type**	**Species**	**Region**	**Examined Factor**	**Findings**
[52]	Case studies	*Scorpaena plumieri* Bloch, 1789 and *Scorpaena brasiliensis* Cuvier, 1829	Brazil	Pain relief	Six patients found pain relief through hot-water immersion. Other methods like systemic painkillers, applying urine, alcohol, and garlic were largely ineffective for pain relief
**Catfish**					
**Reference**	**Research Type**	**Species**	**Region**	**Examined Factor**	**Findings**
[3]	Case studies	suborder *siluroidei*	Western Atlantic Ocean coast	Pain relief	Immersion of affected extremities in hot water was utilised in 20% of patients and was proved beneficial
[23]	Case studies	*Clarias gariepinus* (Burchell, 1822) and *Heteropneustes fossilis* (Bloch, 1794)	Poland	Pain relief	HWI was utilised as pain control treatment
[53]	Case study	*Heteropneustes fossilis* (Bloch, 1794)	Poland	Pain relief	The wound was treated with lidocaine, material removal, and thorough cleansing. The affected hand was immersed in hot water (45 °C) for about 45 min to inactivate venom
[54]	Case study	Unspecified	Iran	Pain relief	Treatment involved immersing the affected limbs in hot water (temperature below 50 °C), which relieved pain within 30 min
**Unspecified/Several Organisms**					
**References**	**Research Type**	**Species**	**Region**	**Examined Factor**	**Findings**
[21]	Review	Marine venomous species (jellyfish, stingray, weeverfish, lionfish, scorpionfish, stonefish etc.)	Unspecified	Pain relief	Employing the highest temperature that the patient can safely endure is optimal
[55]	First part: Retrospective review—phone survey Second part: experimental in vitro studies	Unspecified	Hong Kong, China	Practical solution for the loss of water temperature in the container during HWI	Authors proposed overall recommendations for HWI therapy
[56]	Review	Marine species endemic to the Mediterranean Sea	Mediterranean Sea	Pain relief	Authors recommend HWI at a temperature not exceeding 45 °C for a duration of up to 2 h or until the pain diminishes
[57]	Cross-sectional descriptive study	*Pterois* sp., *Echinoidea*, *Ariidae*, *Chondrichthyes*, *Elapidae*	Australia	Pain relief	In 56.3% cases HWI reduced pain effectively

**Table 2:** Ice-pack treatment summary.

Jellyfish					
Reference	Research Type	Species	Region	Examined Factor	Findings
[12]	Review	*Physalia* sp., *Carybdea alata*, *Carukia barnes*, *Chironex fleckeri*, *Chiropsalmus quadrigatus*, *Chiroplasmus quadrumanus*, *Pelagia noctiluca*, *Cyanea capillata*	Unspecified	Hot water or ice packs	Appropriate envenomation treatment depends on species that caused it
[24]	In vitro, ex vivo	*Alatina alata* and *Chironex flecker*	*C. fleckeri* was collected from Queensland, Australia, meanwhile *A. alata* from Hawaii, USA	Usage of commonly recommended and commercially available methods	Application of ice increased *A. alata* sting severity, while heat was notably effective in reducing hemolytic area for *C. fleckeri*
[58]	Review	Unspecified	Unspecified	Iced seawater or cold packs	Usage of cold water/ice packs demonstrates a potent pain-relieving effect
[59]	Review	*Physalia physalis*, *Cyanea capillata*, *Chironex fleckeri*	Unspecified	Cold and warm water	Warm water is beneficial for bites that do not involve toxins that can be neutralised with heat. Cold water, however, may be more appropriate for severe systemic reactions and reduce pain
[60]	Prospective cohort study	*Chironex fleckeri*	Australia	Vinegar, ice packs and painkillers	Pain of patients could be controlled with vinegar followed by ice treatment
[61]	Randomised controlled trial	*Chironex fleckeri*	Australia	Ice packs and HWI	There was no statistically significant difference between the results of the two treatments
[62]	Retrospective study	Unspecified	Eastern Mediterranean Sea	Various treatment, including ice	Out of 31 patients treated with ice, 21 showed improvement with this treatment.
**Hydroids**					
**Reference**	**Research Type**	**Species**	**Region**	**Examined Factor**	**Findings**
[63]	In vitro studies	*Lytocarpus philippinus*	Australia	Methylated spirits, vinegar, urine, sea water, fresh water, distilled water, *aloe vera* and ‘Stingnose’	Methylated spirits induced discharge in mature mastigophores of the hydroid. Unlike vinegar, it activated nematocysts. Therefore, water dousing followed by ice application is advised for pain relief, consistent with first aid for non-cubozoan jellyfish stings.
**Sea Anemones**					
	**Research Type**	**Species**	**Region**	**Examined Factor**	**Findings**
[19]	Case study	*Anemonia viridis*	Catalonia, Spain	Seawater, vinegar, ammonia, 10% baking soda mixed in seawater, freshwater	Seawater does not trigger cnidocyst activation, making it a neutral rinse solution. Vinegar and ammonia induce significant discharge, while a 10% baking soda solution causes medium discharge. Freshwater leads to low discharge, often leaving undischarged cnidocytes.

## Data Availability

The data, analytical methods, and study materials that support the findings of this study are available from Kamil Jurowski (toksykologia@ur.edu.pl) on reasonable request.
